# Novel Symmetrical Cage Compounds as Inhibitors of the Symmetrical MRP4-Efflux Pump for Anticancer Therapy

**DOI:** 10.3390/ijms22105098

**Published:** 2021-05-12

**Authors:** David Kreutzer, Henry Döring, Peter Werner, Christoph A. Ritter, Andreas Hilgeroth

**Affiliations:** 1 Research Group of Drug Development, Institute of Pharmacy, Martin Luther University Halle-Wittenberg, 06120 Halle, Germany; david.kreutzer94@web.de (D.K.); henry.doering@arcor.de (H.D.); peter.werner@mail.de (P.W.); 2 Department of Clinical Pharmacy, Institute of Pharmacy, Ernst Moritz Arndt University Greifswald, 17489 Greifswald, Germany; ritter@uni-greifswald.de

**Keywords:** anticancer drug, drug resistance, structure activity, synthesis, inhibition

## Abstract

Within the last decades cancer treatment improved by the availability of more specifically acting drugs that address molecular target structures in cancer cells. However, those target-sensitive drugs suffer from ongoing resistances resulting from mutations and moreover they are affected by the cancer phenomenon of multidrug resistance. A multidrug resistant cancer can hardly be treated with the common drugs, so that there have been long efforts to develop drugs to combat that resistance. Transmembrane efflux pumps are the main cause of the multidrug resistance in cancer. Early inhibitors disappointed in cancer treatment without a proof of expression of a respective efflux pump. Recent studies in efflux pump expressing cancer show convincing effects of those inhibitors. Based on the molecular symmetry of the efflux pump multidrug resistant protein (MRP) 4 we synthesized symmetric inhibitors with varied substitution patterns. They were evaluated in a MRP4-overexpressing cancer cell line model to prove structure-dependent effects on the inhibition of the efflux pump activity in an uptake assay of a fluorescent MRP4 substrate. The most active compound was tested to resentisize the MRP4-overexpressing cell line towards a clinically relevant anticancer drug as proof-of-principle to encourage for further preclinical studies.

## 1. Introduction

Cancer remains the second frequent cause of death worldwide, although cancer therapies of the last decades improved by the development of novel drugs and the discovery of novel target structures in cancer cells [1,2,3]. The classical chemotherapeutic treatments with antineoplastic agents affect all dividing cells and have strong side effects [4]. Ongoing resistances enforced the search for novel structures and resulting drugs [5]. With the understanding of the molecular pathways and regulators of cell growth and division the number of potential target structures for drugs increased [3]. Hence, deregulated protein kinases in cancer cells could be identified and X-ray crystal structure analysis helped to find the binding region for potential inhibitors [6]. Novel small-molecule inhibitors were developed and also antibodies were synthesized using biotechnology procedures that address the receptor regions of the respective protein kinases [7]. However, mostly single mutations led to changes in the inhibitor binding protein region and thus cause resistances to the respective drugs [8]. Moreover, the costs for such monoclonal antibody therapies are high and a burden for the social health systems [9]. Therefore, it will be an ongoing challenge to find novel drugs.

As the main cause of anticancer drugs resistance is the occurrence of transmembrane efflux pumps, those structures were early considered as promising target structures [10]. As they reduce the intracellular level of the anticancer drug by outwards transport, the cancer cell becomes resistant [11]. The known cancer-relevant efflux pumps transport various drugs with different structures and thus establish a multidrug resistance (MDR) that can hardly be defeated [11]. Also, the novel more specifically acting anticancer drugs become affected by the MDR phenomenon.

Early inhibitors like verapamil, quinidine or cyclosporine A of efflux pump activity have been evaluated in cancer cells that overexpressed the respective efflux pump to be effective [12]. Partly, they could not be used in clinical trials due to own pharmacological effects like verapamil as calcium antagonist, quinidine as antiarrhythmic agent and cyclosporine A as immunosuppressant drug [12]. Candidates of the third generation with dofequidar, zosuquidar, tariquidar, elacridar, and biricodar were developed as specific efflux pump inhibitors but proved to inhibit also different efflux pumps. In clinical trials they were disappointing because the benefit in tumor therapy was low [11,13,14]. The reason for that was a missing knowledge of an efflux pump expression by the respective tumor [11]. Therefore, recent studies show that in case of a detected expression of an efflux pump the use of such inhibitors is successful [11,15,16].

A strategy to find novel inhibitors or candidates for a further profiling has been the screening of drug libraries [11,12]. Compounds can be identified with their own pharmacological properties that have to be rebuilt to reduce those properties, but maintain the efflux pump inhibition potential [12]. Our strategy was to synthesize novel inhibitors that may be of favor for the inhibition of the symmetric efflux pump multidrug resistant protein (MRP) 4 for that inhibitors are absolutely rare [17]. Similar to the longer known efflux pump P-glycoprotein, MRP4 owns a symmetric molecular structure with two transmembrane domains subdivided in each six *α*-helical units [18,19].

We synthesized cage dimers of two 1,4-dihydropyridines arranged in a *head* to *tail* fashion to provide the favorable *C*_2_ symmetry in the substituent arrangement at the symmetric molecular scaffold. Within the substituents we varied the positioning and number of residues and discuss the results of determined MRP4 inhibition properties. These were determined in a fluorescence uptake assay with a fluorescent MRP4 substrate in two pancreatic cancer cell lines one without expressing MRP4 and one with MRP4 by a fluorescence ratio of the uptaken substrate in both cell lines. Substances with highest ratios and as compared with a standard MRP4 inhibitor characterized best inhibitors. Furthermore, the best inhibitor was evaluated to resensitize the MRP4-overexpressing cell line for a common MRP4 anticancer drug as proof-of-concept.

## 2. Results and Discussion

### 2.1. Formation of the 1,4-Dihydropyridine Cage Dimer Formation

As a reduced pyridine the 1,4-dihydropyridine scaffold owns two neighbored double bonds. A double bond may undergo a [2+2] cycloaddition reaction with another double bond to form cyclobutane compounds after irradiation with light under double bond excitation [20]. Pyrone compounds are known to form photodimers by the reaction of neighbored double bonds under UV light irradiation as cage compounds with resulting neighborhood cyclobutane rings that form the cage compound walls [21]. Such photodimers own a mirror symmetry in case of a *head* to *head* conjunction or a *C*_2_ symmetry in case of a *head* to *tail* conjunction [21,22].

**Scheme 1:**
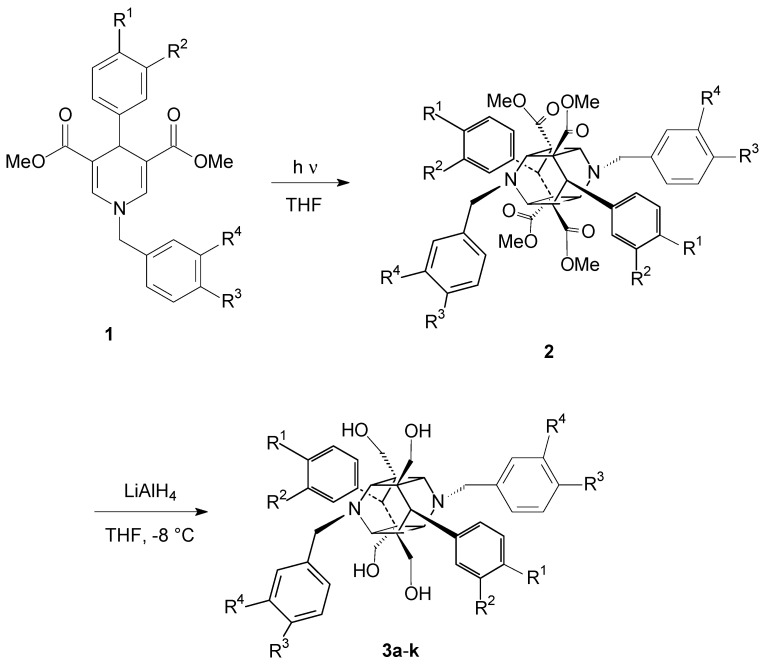
Cage dimer target formation via 1,4-dihydropyridines.

Therefore, we irradiated solutions of synthesized 1.4-dihydropyridines **1** with neighborhood double bonds similar to pyrone compounds to observe the formation of crystallizing cage compounds from the solutions that owned a *head* to *tail* arrangement of the former 1,4-dihydropyridine subunits and thus own a *C*_2_ symmetry of the molecular framework **2** (Scheme 1). The used 1,4-dihydropyridines **1** have been synthesized from an aromatic aldehyde, methyl propiolate and a benzylamine compound in acetic acid as a procedure different from the known Hantzsch synthesis of 1,4 dihydropyridines that use a dicarbonyl compound instead of the methyl propiolate [23].

Those Hantzsch 1,4-dihydropyridines with characteristic alkyl substituents at the 2- and 6-positions of the molecular scaffold are not sensitive to light except of the 1,4-dihydropyridine drug nifedepine as dimethyl 2,6-dimethyl-4-(2-nitrophenyl)- 1,4-dihydropyridine-3,5-dicarboxylate. Nifedipine undergoes a intramolecular redox reaction of the 1,4-dihydropyridine and the nitro function at the 4-phenyl residue to form a pyridine compound [24]. Finally, the four methyl ester residues in compounds **2** have been reduced with lithium aluminium hydride to give alcoholic functions in target compounds **3a**–**k** that were of favor for the compound solubility and may serve as both hydrogen bond acceptors and donators in interaction with amino acid residues of the protein target compound MRP4.

### 2.2. MRP4 Efflux Pump Inhibition with the 1,4-Dihydropyridines

Recent studies prove the role of MRP4 in various kinds of solid cancer like neuroblastoma where it attributes to the poor clinical outcome, pancreatic and prostate cancer and melanoma and myeloid leukemia with effects on cell proliferation and differentiation [11,25,26,27,28]. The number of MRP4 inhibitors is strongly limited so far because the focus on MRP4 as promising target for anticancer therapy is relatively new compared to longer known efflux pumps like P-glycoprotein [25,28,29,30]. Therefore, there is need for novel MRP4 inhibitors. Our successful synthesis provides symmetric molecules that may serve as inhibitors of the symmetric efflux pump MRP4. We introduced methoxy functions in the aromatic residues that have been varied in positioning within the symmetric phenyl residues and in the number. Methoxy functions are long known as favorable substituents for efflux pump inhibition as they may serve as hydrogen bond acceptor functions to amino acid residues of the potential MRP4 binding site [31].

In our assay system, for determination of the MRP4 inhibition we used two pancreatic cancer cell lines one without the expression of MRP4 and the other one with expressing MRP4 after vector plasmid transfection. We determined the uptake of the fluorescent MRP4 substrate calcein in both cell lines each without an inhibitor and with an inhibitor. In case of the inhibition of MRP4 the uptake of calcein is increased due to the lowered outwards transport by the efflux pump. The fluorescence values of each inhibitor-treated cell line are corrected by the fluorescence of the untreated control cells and the resulting values are given as fluorescence activity ratios (*FARs*) of the MRP4-expressing and non-expressing cells, so that the increasing values reflect the degree of MRP4 inhibition. The *FAR* values are shown in Table 1.

We started with compound **3a** without substitution of the aromatic residues. The *FAR* value of 1.15 was much better than that of the used MRP4 inhibitor MK571 with a value of 0.82 as best known standard inhibitor. The introduction of a *para* methoxy function in the *N*-benzyl residues in compound **3b** led to an unchanged activity. The additional introduction of a *para* methoxy function in the phenyl residues increased the activity of compound **3c** to a *FAR* value of 1.34. Then we varied the positioning of the favorable four methoxy functions within the aromatic residues.

If the *para* methoxy functions of the *N*-benzyl residues move to the *meta* positions in compound **3d** the *FAR* value further increased compared to compound **3c**. If the *para* methoxy phenyl functions of compound **3d** also move to the *meta* position in compound **3e** the activity was almost unchanged. If the *meta* methoxy benzyl functions of compound **3e** now move to the *para* positions in compound **3f** best activities were reached with a *FAR* value of 1.70.

We then increased the number of methoxy functions to investigate a suggested increasing effect on activity according to the results with the discussed first compounds. If another methoxy function was introduced in the *N*-benzyl residues of the presently best compound **3f** the activity of derivative **3g** was similar to that of the both *meta* methoxy phenyl and *N*-benzyl substituted compound **3e**. If the *meta* methoxy phenyl function moved to the *para* position of the phenyl residues in compound **3h** the activity was lowered and similar to that of the both *para* methoxy phenyl and *N*-benzyl substituted compound **3c**. If both methoxy functions were concentrated in the phenyl residues of derivative **3i** with a *meta* methoxy *N*-benzyl function the activity was lower than that of compound **3g** with both methoxy functions at the *N*-benzyl residues and the *meta* methoxy function at the phenyl residues. If the *N*-benzyl *meta* methoxy moved to the *para* position in derivative **3j** the activity increased to slightly exceed that of compound **3h** with both methoxy functions at the *N*-benzyl residues and the *para* methoxy function at the phenyl residues. Therefore, it can be stated that those six methoxy functions with a non-symmetrical distribution at the aromantic residues is generally not of favor to increase the activity although increasing numbers of methoxy functions were suggested to be of favor as suggested from the previous compounds.

Then we further increased the number of methoxy functions to eight in compound **3k** with an again symmetric distribution on the aromatic residues. The *FAR* value with 1.74 of that compound was the best one of all tested compounds. Therefore, it can be concluded from the allover results that an increasing number of methoxy functions is of favor for the MRP4 inhibiting properties in case of a symmetric distribution within the symmetrically arranged aromatic residues. The general suggestion that methoxy functions are favorable for the efflux pump inhibiting properties can be confirmed with our study results also for the so far not investigated MRP4. However, the result that the effect is limited to those compounds with a symmetric distribution within the aromatic residues supports the existence of a symmetric binding side or arrangement for inhibitors of that symmetric efflux pump.

### 2.3. In Vitro MRP Resistance Studies of Drug Reversal

In various kinds of solid cancer the role of MRP4 in anticancer drug resistance has been suggested based on mRNA analyses of the MRP4 encoding gene [11]. In order to confirm such observations the expression of MRP4 should be proved. However, the number of MRP4 inhibitors is strongly limited with MK571 as presently best known inhibitor that is a cysteinyl leukotriene receptor antagonist with additional pharmacological properties as phosphodiesterase inhibitor [25,32]. With our evaluated compound class of novel cage dimers **3** we found a tool to evaluate the structure-activity relationships of MRP4 inhibition as discussed.

From that compound class we selected the best inhibitor **3k** for further studies to prove that the MRP4 inhibition effect really contributes to a MRP4 reversal of an anticancer drug resistance as suggested by early publications. We chose 6-mercaptopurin as a known MRP4 substrate that is a commonly used antimetabolite in the chemotherapy of various kinds of cancer. With our MRP4 expressing pancreatic cell line we had a cell model that could be used for the study.

**Figure 1:**
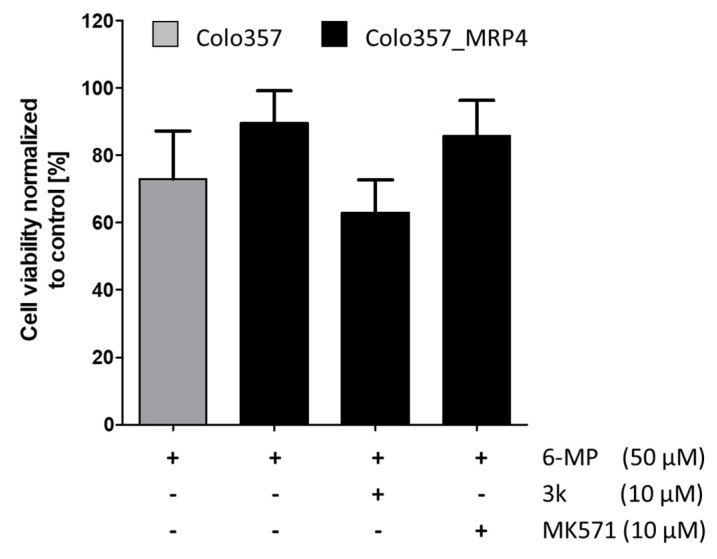
Effect of the symmetric cage compound **3k** on cell viability of MRP4 expressing Colo357 pancreatic cancer cells (Colo357_MRP4). Cells were incubated with 50 µM 6-mercaptopurine (6-MP) in the absence or presence of 10 µM compound **3k** or the unselective MRP4 inhibitor MK571 for 48 h at 37 °C and thereafter cell viability was assessed using an MTT assay as described in the Section 3. Values represent mean ± SEM of three independent experiments. Due to an insolubility of 6-MP at higher concentrations the effect was determined at the given one. Alternatively, the measurement of a concentration-dependent effect and the depiction in complete sigmoid curves was not possible with reliable values.

First we determined the toxicity of 6-mercaptopurin in the non-MRP4 expressing cell line. For that purpose the MTT assay was used to determine the toxicity as degree of a reduced formazan formation from the MTT reagent by the mitochondrial dehydrogenases. In the presence of 50 µM 6-mercaptopurine cell viability of Colo357 cells was reduced to 73 ± 24.7% (mean ± SD, *n* = 3, Figure 1).

Next we determined the 6-mercatopurin toxicity in the MRP4-expressing pancreatic cell line where the toxicity was expected to be much lower due to the MRP4 mediated outwards transport of the drug. We found an only slight reduction of cell viability in Colo357_MRP4 cells to 89.5 ± 16.7% (mean ± SD, *n* = 3, Figure 1) that confirmed the MRP4 efflux pump activity as cause for the lowered 6-mercaptopurin toxicity. Then we used 10 µM of MK571 as potential inhibitor and found a reduction in cell viability to 85.8 ± 18.3% (mean ± SD, *n* = 3, Figure 1) which means no effect on the MRP4 efflux inhibition. Next we used our best inhibitor **3k** with an almost double stronger effect on MRP4 as evaluated *FAR* value. The use of 10 µM of compound **3k** resulted in a 6-mercaptopurin toxicity corresponding to a reduction in cell viability to 63.0 ± 16.9% (mean ± SD, *n* = 3, Figure 1). Thus we observed a marked yet not significant (*p* = 0.125, unpaired *t*-test) effect of a reduced 6-mercaptopurin toxicity for the MRP4-expressing cell line as result of an inhibition of MRP4 as proof-of-principle. An alternative use of naturally MRP4-overexpressing cell lines like the breast cancer cell lines MDA-MB-436 and BT549 was not taken in consideration because those cell lines overexpress also different efflux pumps like P-glycoprotein, BCRP and MRP1 more than MRP4 in MDA-MB-436 and MRP1 and MRP2 in BT549 [29,33,34,35].

## 3. Material and Methods

### 3.1. Chemical Reagents and Instruments

Commercial reagents were used without further purification. The ^1^H-NMR spectra (500 MHz) were measured using tetramethylsilane (Acros Organics, Fair Lawn, NJ, USA) as internal standard. Thin layer chromatography (TLC) was performed on E. Merck 5554 silica gel plates (Merck KGaA, Darmstadt, Germany). The mass spectra were recorded on a Finnigan LCQ Classic mass spectrometer (Thermo Electron, Langenselbold, Germany).

### 3.2. General Procedure for the Synthesis of Compounds ***1***

One equivalent of the aromatic aldehyde, two equivalents of the methyl propiolate (Acros Organics, Fair Lawn, NJ, USA) and one equivalent of the benzylamine were dissolved in 2 mL of freshly distilled acetic acid and heated under reflux and stirring at 100 °C for two hours. After finishing the reaction mixture was cooled down to room temperature and water was added. Then extraction followed with chloroform and was repeated for two times. The unified organcic layer was dried over sodium sulfate and reduced in volume. Then methanol was added under cooling to give the final products.

### 3.3. General Procedure for the Cage Dimer Formation of Compounds ***2***

One and a half equivalent of the respective monomeric 1,4-dihydropyridine **1** was dissolved in dry THF (Sigma Aldrich, St. Louis, MO, USA) in a quartz flask. After fumigating with argon the flask was closed and irradiated with Ultra Vitalux^®^ lamps (Osram, Munic, Germany) that produce light of wavelengths > 270 nm from a distance of 60 cm at room temperature. The reaction was controlled by the use of TLC. A precipitation of the formed cage dimers **2** was observed. After finishing of the irradiation that followed after an observed disappearance of the fluorescent starting compound on the TLC sheets the formed product **2** was filtered off or separated from the solution under cooling and a following filtration procedure. A final recrystallization was carried out from methanol.

### 3.4. General Procedure for the Ester Group Reduction in the Cage Dimers to Form the Target Compounds ***3a**–**k***

Seventy micromole of the respective cage dimer **2** were dissolved in dried THF. After fumigating of the solution with argon it was stirred and cooled down to −8 °C. Then 1.12 mmol of lithium aluminium hydride (Sigma Aldrich, St. Louis, MO, USA) were added dropwise as a one molare solution in THF under stirring for additional three hours at the low temperature. The reaction was followed by the use TLC. After a complete disappearance of the starting compound on the TLC sheet portions of a 20% solution of potassium hydroxide and of ice water were added at a temperature of 0 °C. Then the organic layer was extracted with chloroform for several times. The organic layer was unified, dried over sodium sulfate and filtered. Then the solution volume was reduced under low pressure and a mixture of chloroform, diethyl ether and petrol ether was added dropwise. The precipitated target compounds **3a**–**k** were recrystallized from methanol.

#### 3.4.1. 3,9-dibenzyl-1,5,7,11-tetrakishydroxymethyl-6,12-diphenyl-3,9-diazhexacyclo-[6.4.0.0^2.7^.0^4.11^.0^5.10^]dodecane **3a**

Yield 92%; white solid; mp 226–231 °C; ^1^H NMR (DMSO-d_6_) *δ* 7.79 (d, *J* = 8.0 Hz, 2H, 2-H of phenyl), 7.34–7.29 (m, 10H, benzylic H), 7.27 (d, *J* = 8.0 Hz, 2H, 6-H of phenyl), 7.12, 7.05, 7.03 (t, *J* = 8.0, 8,0, 8,0 Hz, 6H, 3-, 4-, 5-H of phenyl), 4.41 (part X of a ABX system, 4H, CH_2_O*H*), 4.13 (s, 4H, *N*CH_2_), 3.66 (s, 2H, 6-, 12-H), 3.16–3.06 (part AB of the ABX system, 8H, C*H*_2_OH), 2.95 (s, 4H, 2-, 4-, 8-, 10-H); MS (ESI), *m/z* = 615 [M+H^+^]; IR (KBr): 3441, 2930, 1600, 1493 cm^−1^. Anal. (C_40_H_42_N_2_O_4_ × 0.5 H_2_O) Calc. C 77.02, H 6.95, N 4.49; Found C 77,14, H 6.79, N 4.46.

#### 3.4.2. 3,9-bis(4-methoxybenzyl)-1,5,7,11-tetrakishydroxymethyl-6,12-diphenyl-3,9-diazhexa-cyclo[6.4.0.0^2.7^.0^4.11^.0^5.10^]dodecane **3b**

Yield 55%; white solid; mp 197–202 °C; ^1^H NMR (DMSO-d_6_) *δ* 7.81 (d, *J* = 7.6 Hz, 2H, 6-H of phenyl), 7.27–7.24 (m, 4H, 2-, 6-H of benzyl), 7.13–7.00 (m, 6H, 3-, 4-, 5-H of phenyl), 6.87–6.85 (m, 4H, 3-, 5-H of benzyl), 4.40 (part X of a ABX system, 4H, CH_2_O*H*), 4.05 (s, 4H, *N*CH_2_), 3.74 (s, 6H, OCH_3_), 3.65 (s, 2H, 6-, 12-H), 3.11–3.04 (part AB of the ABX system, 8H, C*H*_2_OH), 2.93 (s, 4H, 2-, 4-, 8-, 10-H); MS (ESI), *m/z* = 675 [M+H^+^]; IR (KBr): 3458, 2913, 1607, 1512 cm^−1^. Anal. (C_42_H_46_N_2_O_6_) Calc. C 74.69, H 6.87, N 4.15; Found C 74,45, H 6.75, N 3.95.

#### 3.4.3. 3,9-bis(4-methoxybenzyl)-1,5,7,11-tetrakishydroxymethyl-6,12-bis(4-methoxyphenyl)-3,9-diazhexacyclo[6.4.0.0^2.7^.0^4.11^.0^5.10^]dodecane **3c**

Yield 45%; white solid; mp 238–241 °C; ^1^H NMR (DMSO-d_6_) *δ* 7.70 (d, *J* = 8.7 Hz, 2H, 6-H of phenyl), 7.24 (m, 4H, 2-, 6-H of benzyl), 7.17 (d, *J* = 8.4 Hz, 2H, 2-H of phenyl), 6.88 (m, 4H, 3-, 5-H of benzyl), 6.67 (d, *J* = 8.4 Hz, 2H, 3-H of phenyl), 6.53 (d, *J* = 8.7 Hz, 2H, 5-H of phenyl), 4.36 (part X of a ABX system, 4H, CH_2_O*H*), 4.02 (s, 4H, *N*CH_2_), 3.74, 3.69 (2 × s, 12H, OCH_3_), 3.57 (s, 2H, 6-, 12-H), 3.16–3.05 (part AB of the ABX system, 8H, C*H*_2_OH), 2.89 (s, 4H, 2-, 4-, 8-, 10-H); MS (ESI), *m/z* = 757 [M+Na^+^]; IR (KBr): 3357, 2926, 2835, 1609, 1462, 1246, 1077 cm^−1^. Anal. (C_44_H_50_N_2_O_8_) Calc. C 71.90, H 6.86, N 3.81; Found C 71,55, H 6.95, N 3.75.

#### 3.4.4. 3,9-bis(3-methoxybenzyl)-1,5,7,11-tetrakishydroxymethyl-6,12-bis(4-methoxyphenyl)-3,9-diazhexacyclo[6.4.0.0^2.7^.0^4.11^.0^5.10^]dodecane **3d**

Yield 38%; white solid; mp 203–205 °C; ^1^H NMR (DMSO-d_6_) *δ* 7.01 (d, *J* = 8.6 Hz, 2H, 6-H of phenyl), 7.22–6.85 (m, 4H, 2-, 6-H of benzyl), 6.78 (d, *J* = 8.4 Hz, 2H, 2-H of phenyl), 6.75–6.70 (m, 4H, 3-, 5-H of benzyl), 6.68 (d, *J* = 8.4 Hz, 2H, 3-H of phenyl), 6.52 (d, *J* = 8.6 Hz, 2H, 5-H of phenyl), 4.39 (part X of a ABX system, 4H, CH_2_O*H*), 4.08 (s, 4H, *N*CH_2_), 3.69, 3.64 (2 × s, 12H, OCH_3_), 3.58 (s, 2H, 6-, 12-H), 3.19–3.09 (part AB of the ABX system, 8H, C*H*_2_OH), 2.92 (s, 4H, 2-, 4-, 8-, 10-H); MS (ESI), *m/z* = 735 [M+H^+^]; IR (KBr): 3442, 2915, 2835, 1608, 1455, 1252, 1038 cm^−1^. Anal. (C_44_H_50_N_2_O_8_) Calc. C 71.90, H 6.86, N 3.81; Found C 71.87, H 6.90, N 3.58.

#### 3.4.5. 3,9-bis(3-methoxybenzyl)-1,5,7,11-tetrakishydroxymethyl-6,12-bis(3-methoxyphenyl)-3,9-diazhexacyclo[6.4.0.0^2.7^.0^4.11^.0^5.10^]dodecane **3e**

Yield 32%; white solid; mp 204–206 °C; ^1^H NMR (DMSO-d_6_) *δ* 6.92–6.83 (m 8H, arylic H of benzyl), 7.48–6.62 (m, 8H, arylic H of phenyl), 4.44–4.39 (part X of a ABX system, 4H, CH_2_O*H*), 4.17–4,10 (m, 4H, *N*CH_2_), 3.69, 3.63 (2 × s, 6H, OCH_3_), 3.58 (s, 2H, 6-, 12-H), 3.52, 3.50 (2 × s, 6H, OCH_3_), 3.18–3.09 (part AB of the ABX system, 8H, C*H*_2_OH), 2.96 (s, 4H, 2-, 4-, 8-, 10-H); MS (ESI), *m/z* = 735 [M+H^+^]; IR (KBr): 3428, 2954, 2869, 1597, 1455, 1378, 1259, 1073 cm^−1^. Anal. (C_44_H_50_N_2_O_8_) Calc. C 71.90, H 6.86, N 3.81; Found C 71,65, H 6.75, N 3.82.

#### 3.4.6. 3,9-bis(4-methoxybenzyl)-1,5,7,11-tetrakishydroxymethyl-6,12-bis(3-methoxyphenyl)-3,9-diazhexacyclo[6.4.0.0^2.7^.0^4.11^.0^5.10^]dodecane **3f**

Yield 38%; white solid; mp 206–208 °C; ^1^H NMR (DMSO-d_6_) *δ* 7.25 (m, 4H, 2-, 6-H of benzyl), 6.86 (m, 4H, 3-, 5-H of benzyl), 7.53–6.61 (m, 8H, arylic H of phenyl), 4.39–4.37 (part X of a ABX system, 4H, CH_2_O*H*), 3.93–4,13 (m, 4H, *N*CH_2_), 3.73, 3.71, 3.69 (3 × s, 9H, OCH_3_), 3.57 (s, 2H, 6-, 12-H), 3.49 (s, 3H, OCH_3_), 3.21–3.06 (part AB of the ABX system, 8H, C*H*_2_OH), 2.93 (s, 2H, 2-, 8-H), 2.90 (s, 2H, 4-, 10-H); MS (ESI), *m/z* = 733 [M−H^+^]; IR (KBr): 3376, 2953, 2835, 1607, 1463, 1259, 1073 cm^−1^. Anal. (C_44_H_50_N_2_O_8_) Calc. C 71.90, H 6.86, N 3.81; Found C 71,63, H 6.72, N 3.90.

#### 3.4.7. 3,9-bis(3,4-dimethoxybenzyl)-1,5,7,11-tetrakishydroxymethyl-6,12-bis(3-methoxyphenyl)-3,9-diazhexacyclo[6.4.0.0^2.7^.0^4.11^.0^5.10^]dodecane **3g**

Yield 41%; white solid; mp 200–203 °C; ^1^H NMR (DMSO-d_6_) *δ* 6.87–6.79 (m, 6H, benzylic H), 7.48–6.63 (m, 8H, phenylic H), 4.41–4.36 (part X of a ABX system, 4H, CH_2_O*H*), 4.04 (s, 4H, *N*CH_2_), 3.71, 3.69 (2 × s, 6H, OCH_3_), 3.59 (s, 2H, 6-, 12-H), 3.58, 3.53, 3.49, 3.45 (4 × s, 12H, OCH_3_), 3.21–3.09 (part AB of the ABX system, 8H, C*H*_2_OH), 2.97–2.93 (m, 4H, 2-, 4-, 8-, 10-H); MS (ESI), *m/z* = 817 [M+Na^+^]; IR (KBr): 3536, 2933, 2830, 1603, 1464, 1259, 1027 cm^−1^. Anal. (C_46_H_54_N_2_O_10_) Calc. C 69.50, H 6.85, N 3.52; Found C 69,10, H 6.67, N 3.77.

#### 3.4.8. 3,9-bis(3,4-dimethoxybenzyl)-1,5,7,11-tetrakishydroxymethyl-6,12-bis(4-methoxyphenyl)-3,9-diazhexacyclo[6.4.0.0^2.7^.0^4.11^.0^5.10^]dodecane **3h**

Yield 20%; white solid; mp 204–206 °C; ^1^H NMR (DMSO-d_6_) *δ* 7.71 (m, 2H, AA′-part of the AA′BB′systems, 6-H of phenyl), 7.21 (m, 2H, AA′BB′-part of the AA′BB′system, 2-H of phenyl), 6.86–6.80 (m, 6H, benzylic H), 6.67 (m, 2H, BB′-part of the AA′BB′system, 3-H of phenyl), 6.57 (m, 2H, BB-part of the AA′BB′system, 5-H of phenyl), 4.35 (part X of a ABX system, 4H, CH_2_O*H*), 4.05 (s, 4H, *N*CH_2_), 3.72, 3.69 (2 × s, 6H, OCH_3_), 3.61 (s, 2H, 6-, 12-H), 3.58, 3.49 (2 × s, 12H, OCH_3_), 3.20–3.10 (part AB of the ABX system, 8H, C*H*_2_OH), 2.91 (s, 4H, 2-, 4-, 8-, 10-H); MS (ESI), *m/z* = 817 [M+Na^+^]; IR (KBr): 3536, 2954, 2837, 1607, 1463, 1259, 1072 cm^−1^. Anal. (C_46_H_54_N_2_O_10_) Calc. C 69.50, H 6.85, N 3.52; Found C 69.37, H 6.87, N 3.53.

#### 3.4.9. 3,9-bis(3-dimethoxybenzyl)-1,5,7,11-tetrakishydroxymethyl-6,12-bis(3,4-dimethoxyphenyl)-3,9-diazhexacyclo[6.4.0.0^2.7^.0^4.11^.0^5.10^]dodecane **3i**

Yield 28%; white solid; mp 179–182 °C; ^1^H NMR (DMSO-d_6_) *δ* 6.89–6.80 (m, 8H, benzylic H), 7.40–6.68 (m, 6H, phenylic H), 4.40 (part X of a ABX system, 4H, CH_2_O*H*), 4.08 (s, 4H, *N*CH_2_), 3.70, 3.68, 3.64 (3 × s, 15H, OCH_3_), 3.53 (s, 2H, 6-, 12-H), 3.42 (s, 3H, OCH_3_), 3.24–3.19 (part AB of the ABX system, 8H, C*H*_2_OH), 3.05 (m, 2H, 2-, 8-H), 2.91 (m, 2H, 4-, 10-H); MS (ESI), *m/z* = 817 [M+Na^+^]; IR (KBr): 3478, 2930, 2869, 1599, 1463, 1258, 1073 cm^−1^. Anal. (C_46_H_54_N_2_O_10_) Calc. C 69.50, H 6.85, N 3.52; Found C 69,21, H 6.69, N 3.32.

#### 3.4.10. 3,9-bis(4-methoxybenzyl)-1,5,7,11-tetrakishydroxymethyl-6,12-bis(3,4-dimethoxyphenyl)-3,9-diazhexacyclo[6.4.0.0^2.7^.0^4.11^.0^5.10^]dodecane **3j**

Yield 36%; white solid; mp 206–208 °C; ^1^H NMR (DMSO-d_6_) *δ* 7.23 (m, 4H, 2-, 6-H of benzyl), 6.86 (m, 4H, 3-, 5-H of benzyl), 7.44–6.53 (m, 6H, arylic H of phenyl), 4.36 (part X of a ABX system, 4H, CH_2_O*H*), 4.02 (m, 4H, *N*CH_2_), 3.73, 3.70 (2 × s, 12H, OCH_3_), 3.53 (s, 2H, 6-, 12-H), 3.49 (s, 6H, OCH_3_), 3.16–3.11 (part AB of the ABX system, 8H, C*H*_2_OH), 3.01 (s, 2H, 2-, 8-H), 2.91 (s, 2H, 4-, 10-H); MS (ESI), *m/z* = 817 [M+Na^+^]; IR (KBr): 3487, 2929, 2868, 1609, 1463, 1249, 1073 cm^−1^. Anal. (C_46_H_54_N_2_O_10_) Calc. C 69.50, H 6.85, N 3.52; Found C 69,48, H 6.60, N 3.52.

#### 3.4.11. 3,9-bis(3,4-dimethoxybenzyl)-1,5,7,11-tetrakishydroxymethyl-6,12-bis(3,4-dimethoxy-phenyl)-3,9-diazhexacyclo[6.4.0.0^2.7^.0^4.11^.0^5.10^]dodecane **3k**

Yield 90%; white solid; mp 227–231 °C; ^1^H NMR (DMSO-d_6_) *δ* 6.93–6.80 (m, 6H, arylic H of benzyl), 7.43–6.57 (m, 6H, arylic H of phenyl), 4.36 (part X of a ABX system, 4H, CH_2_O*H*), 4.02, 4.05 (2 × s, 4H, *N*CH_2_), 3.72, 3.71 (2 × s, 12H, OCH_3_), 3.70, 3.68, 3.59 (3 × s, 9H, OCH_3_), 3.54 (s, 2H, 6-, 12-H), 3.49 (s, 3H, OCH_3_), 3.20–3.12 (part AB of the ABX system, 8H, C*H*_2_OH), 3.01 (s, 2H, 2-, 8-H), 2.91 (s, 2H, 4-, 10-H); MS (ESI), *m/z* = 877 [M+Na^+^]; IR (KBr): 3323, 2926, 2835, 1594, 1463, 1255, 1072 cm^−1^. Anal. (C_42_H_58_N_2_O_12_) Calc. C 67.43, H 6.84, N 3.28; Found C 67.24, H 6.60, N 3.22.

### 3.5. MRP4 Inhibition Assay

The human pancreatic carcinoma cell lines colo357 and colo357_MRP4 were used. Colo357_MRP4 cells were generated by transfection of the colo357 cells with a pcDNA3.1-Hygro/MRP4 construct described in [36] using FuGENE transfection reagent (Roche, Mannheim, Germany) according to the manufacturer’s protocol. After 48 h, the cells were split, and stable transfectants were selected using medium containing hygromycin B. MRP4 overexpression was confirmed by Western blotting after separating proteins on a 8% polyacrylamide gel, transfer to nitrocellulose membranes and detecting MRP4 protein using the MRP4 rabbit polyclonal antiserum SNG [36] in a 1:1000 dilution followed by a goat anti-rabbit-IgG HPR-conjugate at a 1:2000 dilution as shown in the Supplementary Materials. Both cell lines were cultured in RPMI-1640 medium that was supplemented with fetal bovine serum (10%) and penicillin/streptomycin (1%) at 37 °C and under carbon dioxide atmosphere (5%).

In the assay, each 200.000 cells were given in an Eppendorf tube. The cells were centrifuged at 2000 rpm at 4 °C. The supernatant was removed and the samples were stored on ice. Then they were resuspended in RPMI-1640 medium and test compounds and MK571 control were added from stock solutions of 4000 µM in DMSO to give a final concentration of 10 µM. The samples were cultured at 37 °C for 20 min and 1200 rpm in a thermomixer. Then the fluorescent calcein was added from a PBS solution to give a final concentration of 0.005 µM. The samples were centrifuged again and the supernatant was removed. Then PBS was added and the samples were centrifuged again. That washing procedure was repeated. Finally, the fluorescence of the resuspended cells was measured by flow cytometry using 10.000 cells and a MACSQuant Analyzer (Miltenyi Biotec, Bergisch Gladbach, Germany). The measurement was conducted each for three times for inhibitor treated and untreated cells of both cell lines.

The *FAR* values were calculated as ratio of the fluorescence of the treated colo357_MRP4 to the tretated colo357 cells with each values corrected by the fluorescence of the untretated colo cells.

### 3.6. MTT-Based Viability Assay

10.000 cells of each cell line were cultured in wells of a 96-well plate at 37 °C under a carbon dioxide atmosphere (5%) for 24 h. Then 50 µM of the MRP4 substrate 6-mercaptopurin, the test compound and MK571, each in a 10 µM final concentration were added. The plate was incubated for 48 h under the original culture conditions. Then the MTT reagent was added to each well (10 µL of a stock solution of 5 mg/mL in PBS) and incubation continued for 4 h. Then 100 µL DMSO were added to each well to solve the formazan reduction product. The plate was shaken for 30 min on a plate shaker and finally the formazan absorption was measured at 570 nm and 630 nm as reference wave length on an Infinite 200 Pro plate reader (Tecan Group Ltd., Männedorf, Switzerland). For further analyses, reference values were subtracted from primary values at 570 nm and resulting values were normalized to values of cells treated with the solvent DMSO, which represent 100% cell viability. Measurements were repeated three times independently, mean ± SD of triplicate values were calculated and statistical analysis was performed using GraphPadPrism version 5.02 (GraphPad Software, San Diego, CA, USA).

## 4. Conclusions

Due to ongoing resistances against anticancer drugs the challenge to combat cancer is the development of novel anticancer drugs that affect novel target structures. The expression of transmembrane efflux pumps mostly under therapy becomes a central problem as they may also transport those novel drugs out of the cells and thus constitute the multidrug resistance. The discovery to find effective inhibitors of such efflux pumps would mean a great progress for anticancer therapy.

We discovered a novel class of symmetric cage compounds that effectively inhibits the activity of the symmetric efflux pump MRP4. As only few inhibitors of MRP4 are known an insight in structure–activity relationships of MRP4 inhibitors has not been possible so far. Our compound class gives first information that methoxy functions are of favor for the inhibitory properties. Increasing numbers of such methoxy functions enlarge the activity in case of a symmetric distribution within the symmetrically arranged aromatic residues. Those results suggest a symmetric binding of the inhibitors to a so far unknown binding region. Additionally, one best compound was able to resensitise the MRP4-expressing subline to the used anticancer drug. These results encourage for further preclinical studies to combat the cause of the MDR in cancer.

## Figures and Tables

**Table 1:** MRP4 inhibition data of target compounds **3a**–**k** with varied substitution patterns expressed as *FAR* values.

Cpd.	R^1^	R^2^	R^3^	R^4^	*FAR* Value ^a^
**3a**	H	H	H	H	1.15
**3b**	H	H	OMe	H	1.15
**3c**	OMe	H	OMe	H	1.34
**3d**	OMe	H	H	OMe	1.56
**3e**	H	OMe	H	OMe	1.52
**3f**	H	OMe	OMe	H	1.70
**3g**	H	OMe	OMe	OMe	1.55
**3h**	OMe	H	OMe	OMe	1.36
**3i**	OMe	OMe	H	OMe	1.25
**3j**	OMe	OMe	OMe	H	1.41
**3k**	OMe	OMe	OMe	OMe	1.74
MK571					0.82

^a^ Mean of three determinations.

## Data Availability

Additional data are available from the authors on request.

## Supplementary Materials

The following are available online at https://www.mdpi.com/article/10.3390/ijms22105098/s1. The following are available online, Western blot of MRP4 overexpression.
