# Supplementary material for: Novel Symmetrical Cage Compounds as Inhibitors of the Symmetrical MRP4-Efflux Pump for Anticancer Therapy
Source: Int J Mol Sci. 2021 May 12;22(10):5098. doi: 10.3390/ijms22105098 (PMC8150856; doi:10.3390/ijms22105098)

Supplementary Materials

Communication

Novel symmetrical cage compounds as inhibitors of the symmetrical MRP4-efflux pump for anticancer therapy

David Kreutzer [1], Henry Döhring [1], Peter Werner [1], Christoph Ritter [2] and Andreas Hilgeroth [1,*]

[1] Institute of Pharmacy, Research Group of Drug Development, Martin Luther University Halle-Wittenberg; andreas.hilgeroth@pharmazie.uni-halle.de

[2] Institute of Pharmacy, Department of Clinical Pharmacy, Ernst Moritz Arndt University Greifswald; ritter@uni-greifswald.de

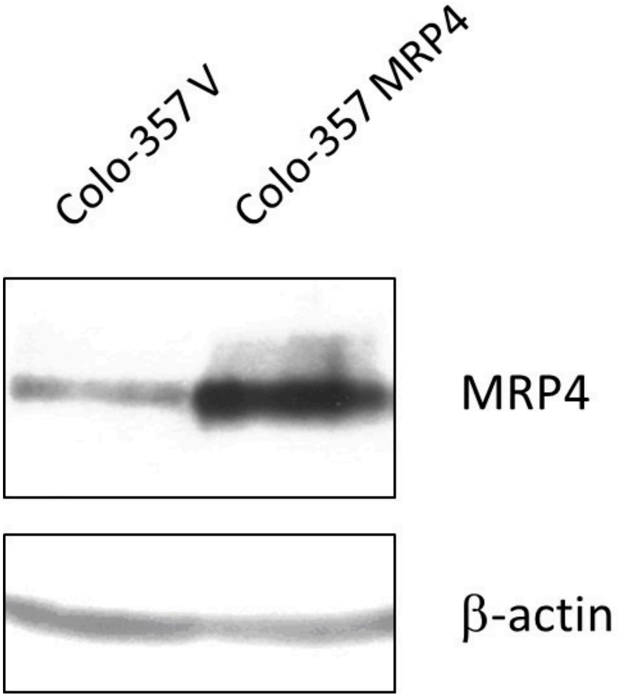

Supplement: Supplementary file 1 [file ijms-22-05098-s001.zip › ijms-1152095-supplementary.pdf]
